# The Acceptability of Pharmacy-Based HPV Vaccination in Western Kenya among Pharmacy Clients and Providers

**DOI:** 10.3390/vaccines11121808

**Published:** 2023-12-02

**Authors:** Shengruo Zhang, Benn Kwach, Victor Omollo, Magdaline Asewe, Rachel C. Malen, Parth D. Shah, Josephine Odoyo, Nelly Mugo, Kenneth Ngure, Elizabeth A. Bukusi, Katrina F. Ortblad

**Affiliations:** 1 Department of Epidemiology, University of Washington, Seattle, WA 98195, USA; 2 Centre for Microbiology Research, Kenya Medical Research Institute, Nairobi 00200, Kenya; kwachbenn@gmail.com (B.K.); victorciv2@gmail.com (V.O.); maggysewe@gmail.com (M.A.); josodoyo@gmail.com (J.O.);; 3 Public Health Sciences Division, Fred Hutchinson Cancer Center, Seattle, WA 98109, USA; rmalen@fredhutch.org (R.C.M.); pshah@fredhutch.org (P.D.S.); kortblad@fredhutch.org (K.F.O.); 4 Center for Clinical Research, Kenya Medical Research Institute, Nairobi 00200, Kenya; rwamba@uw.edu; 5 Department of Global Health, University of Washington, Seattle, WA 98195, USA; 6 School of Public Health, Jomo Kenyatta University of Agriculture and Technology, Nairobi 00200, Kenya; kngure@pipsthika.org; 7 Department of Obstetrics and Gynecology, University of Washington, Seattle, WA 98195, USA

**Keywords:** HPV vaccine, Africa, pharmacy, acceptability, cost

## Abstract

Vaccine coverage for the human papillomavirus (HPV) remains low globally, and differentiated models of vaccine delivery are needed to expand access. Pharmacy-based models of the HPV vaccination may engage women who could benefit. We assessed the acceptability of such a model among pharmacy clients and providers at 20 private pharmacies in Kisumu County, Kenya. In questionnaires, participants (≥18 years) were asked the extent they agreed (5-point scale) with statements that assessed different acceptability component constructs outlined in the Theoretical Framework of Acceptability (TFA). From March to June 2022, 1500 pharmacy clients and 40 providers were enrolled and completed questionnaires. Most clients liked the intervention (TFA: affective attitude; 96%, 1435/1500) and did not think it would be hard to obtain (TFA: burden; 93%, 1399/1500). All providers agreed the intervention could reduce HPV infection (TFA: perceived effectiveness) and felt confident they could deliver it (TFA: self-efficacy). Among the clients who had received or were planning to receive the HPV vaccine in the future, half (50%, 178/358) preferred a pharmacy-based HPV vaccination. In this study, most Kenyan pharmacy clients and providers perceived a pharmacy-delivered HPV vaccination as highly acceptable; however, more research is needed to test the feasibility and effectiveness of this novel vaccine delivery model in Africa.

## 1. Introduction

Human papillomavirus, or HPV, causes most cervical, vaginal, anal, penile, and oropharyngeal cancers globally [1]. One of the main preventive strategies to lessen the excessive burden of HPV-related cancers and diseases is HPV vaccination [2]. There is robust evidence that HPV vaccinations protect against HPV types 16/18, the main causes of invasive cervical cancer, in Kenya [3].

Coverage of the highly effective HPV vaccine remains low among young women in many sub-Saharan African countries, including Kenya [4,5]. In Kenya, HPV vaccination coverage among girls < 15 years of age (i.e., the target population for vaccination delivery) was 33% in 2020 (with only 16% having received their second dose) [6], far below the global target of 90% coverage by 2030 [7]. To help expand vaccination coverage, many African countries, including Kenya, have tried integrating HPV vaccination into free childhood immunization [8] and school-based HPV vaccination programs [9,10,11]. However, challenges with uptake remain, including a lack of knowledge and awareness about HPV [12,13], low HPV vaccination availability and accessibility in the region [14], vaccine hesitancy among the parents of adolescents, and disinformation about vaccines [6]. New and innovative HPV vaccine delivery strategies are needed to reach populations at risk of infection, create equitable access to vaccination, and ultimately reduce the burden of cervical cancer in Africa and globally.

Private pharmacies, which are ubiquitous and often outnumber healthcare facilities in Africa, may be well-suited to deliver the HPV vaccine. In many low- and middle-income countries, individuals often first seek healthcare services at private pharmacies and then visit public healthcare facilities if their health issues remain unresolved [15,16,17,18,19,20]. Pharmacy clients in Kenya have reported a preference for pharmacy-delivered versus clinic-delivered health services for reasons including increased convenience, privacy, respect, and drug access [21]. The potential for private pharmacies to expand the reach of health services, including vaccinations, is becoming increasingly recognized. In several high-income countries, pharmacies played a key role in expanding access to the COVID-19 vaccine during the pandemic [22] and pharmacies are increasingly being recognized and used to deliver other vaccinations, like HPV vaccination, in the United States [23].

To understand the acceptability of delivering the HPV vaccine at private pharmacies in Kenya, we conducted surveys with pharmacy providers and clients. This study could inform the development of a new HPV vaccination service delivery strategy that uses existing health infrastructure to reach individuals who could benefit from an HPV vaccination and are already seeking sexual and reproductive health services at private pharmacies.

## 2. Materials and Methods

### 2.1. Study Design and Setting

We conducted a cross-sectional survey with pharmacy providers and clients in Kenya. Specifically, we engaged 20 private, community-based pharmacies in Kisumu County (western Kenya). Kisumu County has an urban and semi-urban population and 119 licensed community pharmacies as of 2022 [24]. These pharmacies were participating in a study assessing the performance of blood-based HIV self-testing compared to HIV rapid diagnostic testing [25]. Pharmacies were eligible for study participation if they had a full-time licensed pharmacist or pharmaceutical technologist, a private room for HIV testing and associated counseling services, and were willing to participate in research activities, including having a research assistant stationed at the pharmacy full-time to complete questionnaires with pharmacy clients and providers.

### 2.2. Study Participants

We recruited pharmacy clients and providers at the participating pharmacies to engage in research activities. Eligible pharmacy clients were ≥18 years old, reported a recent sexual behavior associated with the risk of HIV acquisition, and were willing to engage in research activities [25]. Because HPV and HIV co-infection is quite high in sub-Saharan Africa, we wanted to learn about this population’s perspective about obtaining HPV vaccination in community pharmacy settings. To determine behaviors associated with HIV risk, we used a modified version of Kenya’s 8-item HIV Rapid Assessment Screening Tool, which asks individuals to self-report different sexual behaviors in the past six months (e.g., condomless sex, engagement in transactional sex); this tool is commonly used at public healthcare clinics in Kenya to determine eligibility to HIV prevention services, including pre-exposure prophylaxis [26]. Eligible pharmacy providers were ≥18 years old, registered pharmacists or pharmaceutical technologists, employed by one of the participating pharmacies, and willing to engage in research activities.

All research procedures were approved by the Scientific Ethics Review Unit at the Kenya Medical Research Institute (Nairobi, Kenya, IRB# 4357) and the Institutional Review Board at the Fred Hutchinson Cancer Center (Seattle, WA, USA, IRB# 10797). All participants completed written informed consent (available in English, Dholuo, and Kiswahili) with a trained Kenyan research assistant and were compensated 500 Kenyan Shillings (~USD 4.40) for their time completing research activities.

### 2.3. Data Collection

Research assistants completed questionnaires with pharmacy clients and providers. All questionnaires were administered in-person in private rooms at pharmacies. The client questionnaire assessed participants’ demographics (e.g., age, sex, education, relationship status), contraception use, sexual behaviors associated with the risk of HPV acquisition (e.g., multiple sexual partners), and mental health status. Additionally, the client questionnaire assessed participants’ HPV knowledge, HPV vaccination history, and interest in the HPV vaccine (described below). The provider questionnaire assessed participants’ demographics (e.g., age, sex, education) and work history (e.g., length of time in the profession, days worked per week). Both the client and provider questionnaires assessed participants’ perceived acceptability of HPV vaccination at the pharmacy and their willingness to pay for or provide the HPV vaccine in this setting (described below). All data were collected electronically via tablets using CommCare (Dimagi, Cambridge, MA, USA).

### 2.4. HPV Knowledge and Vaccination History/Interest

Clients reported their knowledge of HPV, cervical cancer, and the HPV vaccine. Specifically, they reported if they had ever heard of HPV or genital warts, ever heard of cervical cancer, or knew someone with cervical cancer, and ever heard of the HPV vaccine or received a dose of the vaccine. Clients who had received the HPV vaccine or who had heard of HPV vaccination and were interested in getting it in the future were additionally asked to report their preferred setting for HPV vaccination access; response options included public clinics, private clinics, private pharmacies, and online/at-home delivery.

### 2.5. Acceptability Assessment

To assess clients’ and providers’ perceived acceptability of HPV vaccination at private pharmacies, we asked them how strongly they agreed or disagreed (using a 5-point Likert scale) with statements that assessed different acceptability component constructs outlined in the Theoretical Framework for Acceptability (TFA) [27]. The TFA defines acceptability as a multi-dimensional concept with the following seven component constructs: affective attitude, burden, perceived effectiveness, ethicality, intervention coherence, opportunity costs, and self-efficacy [27]. The statements we used for acceptability assessment were adapted from ones suggested by the authors of the TFA [28] and refined with input from local research staff and through pilot testing (to ensure they were comprehendible and measuring what we intended). If >80% of participants reported agreeing or strongly agreeing to a particular statement (or disagreeing or strongly disagreeing with statements that were reverse coded), we determined that the component construct was perceived acceptable [29].

### 2.6. Willingness to Pay Assessment

In our questionnaires, we assessed the amount that clients and providers were willing to pay for or provide an HPV vaccination at a pharmacy (i.e., cost per injection visit). For providers, we asked them to assume they receive the vaccines for free (i.e., from a public-sector payer like the Ministry of Health) and to just consider costs associated with provider time (for client counseling, injection delivery, and record-keeping) and resources (for vaccine storage and delivery supplies). Additionally, we asked providers to report the minimum and maximum price they would charge for pharmacy-based HPV vaccination delivery.

### 2.7. Analysis

We used descriptive statistics to report HPV knowledge, acceptability, and willingness to pay outcomes for all participants. For HPV knowledge and willingness to pay outcomes, we additionally reported client outcomes by women < 25 years, men < 25 years, women ≥ 25 years, and men ≥ 25 years old. To determine if there were any significant differences (*p* < 0.05) in the proportions between subgroups of interest, we used chi-square tests. To understand client demographics associated with a preference for receiving the HPV vaccination at a pharmacy (among clients that reported this outcome), we conducted correlation analyses among all pharmacy clients and stratified analyses by subgroups of male and female clients. Stata v14.0 (College Station, TX, USA) was used for all analyses.

## 3. Results

From March to June 2022, we screened 1691 pharmacy clients and enrolled 1500. Additionally, we enrolled 40 pharmacy providers (median: 2 per pharmacy, interquartile range [IQR] from 1 to 2.75). Among the 1500 clients, 64% (954/1500) were women and the median age was 26 years (IQR 22–31), Table 1. Among the 40 providers, 40% (16/40) were female, 42% (17/40) were pharmacy owners, and the median time in the profession was 6 years (IQR from 4 to 10).

### 3.1. HPV Knowledge and Vaccination Coverage

**Figure 1:**
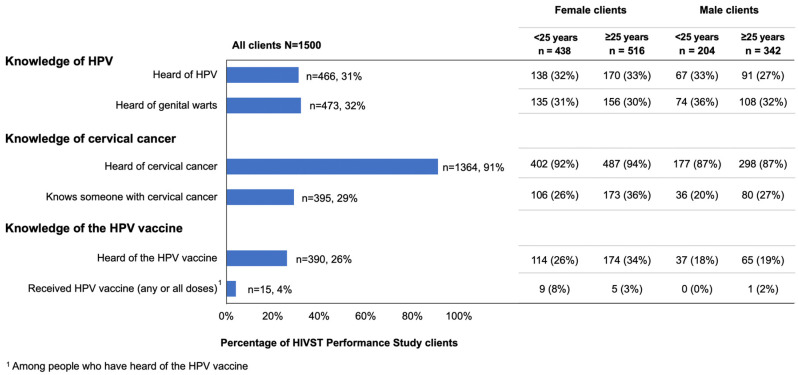
Knowledge of HPV, cervical cancer, and the HPV vaccine.

Among pharmacy clients, knowledge of cervical cancer was high, but knowledge of HPV and the HPV vaccine was less common, Figure 1. While almost all clients (91%, 1364/1500) had heard of cervical cancer, only a third (31%, 466/1500) had heard of HPV, and only a quarter (26%, 390/1500) had heard of the HPV vaccine. Knowledge of the HPV vaccine was higher among women (30%, 288/954) compared to men (19%, 102/546, *p* < 0.001) and highest among women ≥ 25 years (34%, 174/516). Few clients (4%, 15/390) who had head of the HPV vaccine had received at least one vaccination dose, a finding that remained consistent even among the subgroup of women < 25 years (8%, 9/114). Among clients who had heard of but not received the HPV vaccine, most (92%, 343/375) were interested in receiving the vaccine in the future, and this interest was significantly higher among women (96%, 262/274) compared to men (80%, 81/101, *p* < 0.001).

### 3.2. Acceptability of Pharmacy-Based HPV Vaccination

**Figure 2:**
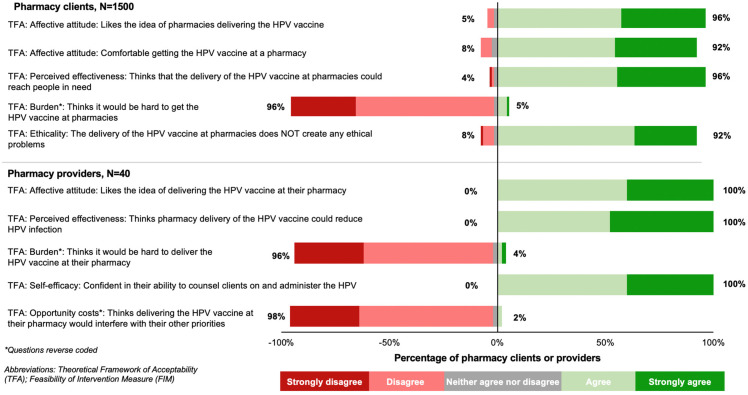
The acceptability of private pharmacies delivering the HPV vaccine among pharmacy clients and providers.

Among pharmacy clients and providers, HPV vaccination at private pharmacies was perceived as highly acceptable (Figure 2). Most clients liked the idea of pharmacies delivering the HPV vaccine (96%, 1435/1500; TFA: affective attitude), felt comfortable receiving an HPV vaccine at a pharmacy (92%, 1375/1500; TFA: affective attitude), and thought that an HPV vaccination at the pharmacy would reach people in need (96%, 1444/1500; TFA: perceived effectiveness). Additionally, few clients thought it would be hard to receive an HPV vaccine at the pharmacy (5%, 71/1500; TFA: burden) and most agreed that receiving an HPV vaccine at a pharmacy would not create any moral or ethical problems (92%, 1379/1500; TFA: ethicality).

All pharmacy providers liked the idea of pharmacies delivering the HPV vaccine (TFA: affective attitude) and agreed that having the HPV vaccine available at pharmacies would be a good way to reduce HPV infection in the community (TFA: perceived effectiveness; Figure 2). Additionally, all pharmacy providers felt confident in their ability to counsel on and administer the HPV vaccine (TFA: self-efficacy). Two providers thought it would be hard to deliver the HPV vaccine at the pharmacy (TFA: burden) and one felt that delivering HPV vaccines at a pharmacy would interfere with their other priorities (TFA: opportunity costs).

### 3.3. Willingness to Pay for HPV Vaccination at Pharmacies

**Figure 3:**
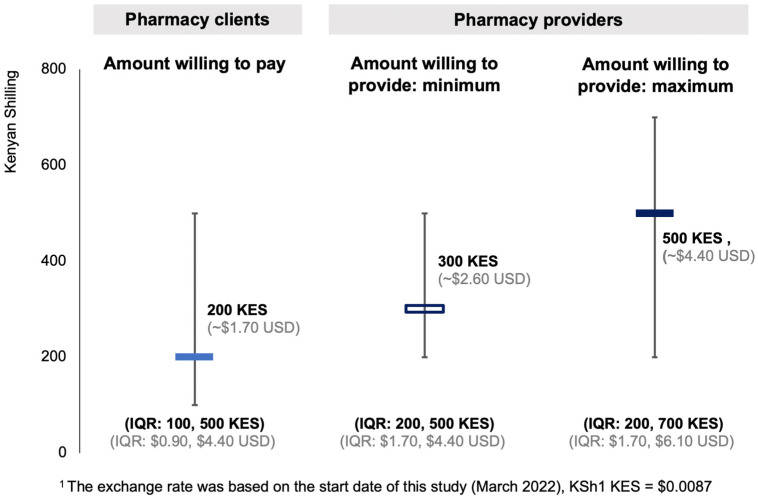
Amount willing to pay for or provide the HPV vaccine at pharmacies among clients and providers ^1^.

Pharmacy clients and providers were willing to pay for or provide HPV vaccination at private pharmacies, Figure 3. Most clients (98%, 1463/1500) reported that they would be willing to pay for an HPV vaccination at private pharmacies; the median amount they were willing to pay was 200 Kenya Shillings (KES; IQR from 100 to 500 KES) or ~USD 1.70 (USD; IQR from ~USD 0.90 to ~USD 4.40). There was no significant difference in the amount clients that were willing to pay for a pharmacy-delivered HPV vaccination between male and female clients nor between clients <25 years and ≥25 years old. All pharmacy providers reported they would be willing to provide the vaccine; the minimum amount they would be willing to charge clients for HPV vaccination was 300 KES (IQR from KES 200 to KES 500) or ~USD 2.60 (IQR from ~USD 1.70 to USD 4.40) and the maximum was 500 KES (IQR from KES 200 to KES 700) or ~USD 4.40 (IQR from ~USD 1.70 to USD 6.10).

### 3.4. Preferred Setting for HPV Vaccination

When clients who reported receiving the HPV vaccine or being interested in receiving it in the future were asked about their preferred setting for HPV vaccination, roughly half (50%, 178/358) reported a preference for receiving the HPV vaccine at a private pharmacy. The next most preferred setting for HPV vaccination delivery was a public clinic (38%, 136/358), followed by a private clinic (11%, 38/358) and online/at-home delivery (2%, 6/358). In the correlation analysis, after adjusting for age, sex assigned at birth, education, and number of children, we did not find any client characteristics significantly associated with a preference for HPV vaccination at a private pharmacy among all clients, female clients, and male clients.

## 4. Discussion

In our study, a pharmacy-delivered HPV vaccination was perceived as highly acceptable among Kenyan pharmacy clients and providers, even when HPV vaccination coverage among pharmacy clients interviewed (including among women < 25 years old) was low, presenting a potential opportunity for intervention. Among clients who reported receiving or being interested in receiving the HPV vaccine in the future, there was great interest in HPV vaccination access at private pharmacies. Most pharmacy clients reported that they would be willing to pay for a pharmacy-based HPV vaccination, however, the amount clients were willing to pay was below the minimum amount the pharmacy providers were willing to provide for HPV vaccination.

While HPV vaccination coverage was low in this population of pharmacy clients, many unvaccinated clients were interested in receiving the HPV vaccine, indicating a potential opportunity for pharmacy-based HPV vaccination to reach underserved populations in need. The population surveyed in this study (i.e., individuals ≥ 18 years old), however, is not the focus of Kenya’s national HPV vaccination program, which primarily targets adolescent girls and young women from 9 to 15 years old [9]. Other populations, including late adolescents and young adults who are or about to become sexually active, could also benefit from an HPV vaccine. Men are another important population that could benefit from an HPV vaccine, as HPV vaccination both decreases the risk of HPV-associated cancers in men (i.e., oral, pharyngeal, anal, and genital cancers) [30,31] and HPV transmission to their sexual partners; gender-neutral HPV vaccination is available in a number of countries including the United States, Canada, Australia, and Switzerland [32,33]. Private pharmacies provide an optional and highly acceptable avenue for HPV vaccine delivery, especially among young women and men who may have missed the vaccine provided at ages from 9 to 15 years in Kenya.

For a model of a pharmacy-based HPV vaccination to be scaled, HPV vaccinations need to be delivered at a price that is affordable to clients in need and still incentivizing and profitable to pharmacy providers. Furthermore, the pharmacy clients who could potentially benefit the most from a pharmacy-based HPV vaccination (e.g., late adolescents and young adults) might be those who face the greatest challenges paying for pharmacy-based services. To make pharmacy-based HPV vaccination accessible to all in need, vaccination vouchers for pharmacy clients could be considered [34] as well as cash incentives given directly to pharmacy providers for service delivery [35], with potentially larger incentives for HPV vaccination delivery to clients <25 years. Both of these interventions, however, might require support from a public-sector pay or donor unless economic analyses can demonstrate an increased profit for pharmacy providers in other areas with the provision of HPV vaccination delivery (i.e., from increased client volume or sales of other services at clients’ HPV vaccination visit).

Additional challenges to the scale-up of this pharmacy-based HPV vaccination model in Kenya include a lack of training on vaccination service delivery among pharmacy providers, affordable vaccinations for pharmacy distribution, and supporting infrastructure (e.g., qualified refrigerators) for vaccine storage and delivery at the pharmacies. To address these challenges, a curriculum for pharmacy providers could be developed to train providers on the delivery of safe injections, screen for potential adverse vaccination events, and deliver associated counseling services. This curriculum could be either incorporated into a certificate program for pharmacy providers or incorporated into continuing education courses or training programs in pharmacy schools (as is performed in Australia) [36]. For access to affordable vaccines that could be distributed at a price accessible to clients, public–private partnerships could also be established to deliver government or donated vaccines at private pharmacies, or collaborations with generic pharmaceutical companies could be developed for access to more affordable vaccines.

This study’s strengths include a large sample of pharmacy clients and providers, helping with the generalizability of study findings among the Kenyan general population and pharmacy providers. Additionally, our study is guided by a widely used theory on service acceptability and used instruments tested in the general Kenyan population. We also acknowledge the limitations of our study. First, all our study outcomes were self-reported and thus subject to social desirability bias, which could have inflated our acceptability outcomes if clients reported more desirable attitudes towards pharmacy-based HPV vaccination when surveyed. However, we tried to mitigate this bias by conducting these surveys in a private room of the pharmacy with trained study staff (as opposed to pharmacy personnel administering the survey). Second, since pharmacy-based HPV vaccination is not currently happening in Kenya, and many pharmacy clients were learning about HPV and the HPV vaccination for the first time, participants were asked to provide their attitudes on a hypothetical client care service. Third, we only asked about interest in HPV vaccination among pharmacy clients who have heard of the HPV vaccine but not received it, which was a missed opportunity to assess interest in the HPV vaccination among all pharmacy clients, including those learning of HPV and the HPV vaccination for the first time. Fourth, the population surveyed did not include individuals from 9 to 15 years old—the target age band of Kenya’s national HPV program. More research is needed to assess the acceptability of pharmacy-based HPV vaccination in this younger population and whether pharmacy-based HPV vaccination is more suited for non-school-age or catch-up HPV vaccination clients [37]. Finally, private pharmacies are diverse settings in Kenya in terms of the clientele served, prioritization of services delivered, and quality of service delivery. As such, participants’ perceptions of the intervention and willingness to pay for or provide the intervention could have been impacted by the characteristics of the pharmacy in which they were being surveyed.

## 5. Conclusions

The delivery of the HPV vaccine at private pharmacies was perceived as highly acceptable among pharmacy clients and providers in Kenya. This novel delivery model may help reach populations that could benefit and are being missed with existing national HPV vaccination campaigns, ultimately contributing to a reduced incidence of HPV-associated cancers, including cervical cancer [38], for which the burden is greatest among women living in sub-Saharan Africa [5]. Before this model can be scaled, however, more research on the feasibility, costs, and sustainability of pharmacy-delivered HPV vaccination in Kenya is needed. Additionally, a curriculum for training pharmacy providers on vaccination delivery needs to be developed, as well as a strategy for supporting the infrastructure needed for vaccine delivery at private pharmacies. If a successful care pathway for a pharmacy-based HPV vaccination delivery in Kenya is developed, this could serve as an example for other countries in the region as well as related interventions, including other vaccinations, injectable contraceptive methods, and potentially long-acting injectable forms of HIV pre-exposure prophylaxis and treatment [39]. This study generated preliminary evidence about the acceptability of community pharmacies that could be helpful for policymakers thinking about alternative settings for expanding HPV vaccination. Ultimately, this novel HPV vaccination delivery approach could also bring us closer to achieving the World Health Organization’s global Cervical Cancer Elimination Initiative, which aims to eliminate cervical cancer within the next century [7].

## Figures and Tables

**Table 1:** Characteristics of the enrolled clients and pharmacy providers.

Clients	All Clients (N = 1500)	Female Clients (N = 954)	Male Clients (N = 546)
Age, med (IQR)	26 (22, 31)	25 (22, 30)	27 (23, 34)
Age < 25 years	642 (43%)	438 (46%)	204 (37%)
Sex—Female	954 (64%)	954 (100%)	0 (0%)
Years in school, med (IQR)	14 (11, 16)	13 (11, 15)	14 (12, 16)
Monthly household income (In Kenyan Shillings), med (IQR)	10,000 (5000, 20,000)	10,000 (5000, 20,000)	10,000 (5375, 20,000)
Relationship Status			
One primary partner only	794 (53%)	583 (61%)	211 (39%)
One primary partner and casual partners	351 (23%)	164 (17%)	187 (34%)
Casual partner(s) only	276 (18%)	156 (16%)	120 (22%)
Single	75 (5%)	49 (5%)	26 (5%)
Other	4 (0%)	2 (0%)	2 (0%)
Period of traveling from home to pharmacy			
<5 min	275 (18%)	156 (16%)	119 (22%)
5 to <15 min	538 (36%)	339 (36%)	199 (36%)
15 to <30 min	481 (32%)	327 (34%)	154 (28%)
≥30 min	206 (14%)	132 (14%)	(14%)
Age of first sex, med (IQR)	17 (15, 18)	17 (16, 19)	17 (15, 18)
Number of living children, med (IQR)	1 (0, 2)	1 (0, 2)	1 (0, 2)
Have ever been pregnant (Female only)	-	692 (73%)	-
Have used EC more than twice (Female only)	-	393 (41%)	-
Using a birth control method (Female only) ^1^	-	749 (79%)	-
Sexual behaviors associated with HPV risk			
Multiple sexual partners ^2^	510 (34%)	250 (26%)	260 (48%)
Inconsistent condom use ^3^	1310 (87%)	845 (89%)	465 (85%)
Exchanged sex for money/gift ^3^	285 (19%)	158 (17%)	127 (23%)
Diagnosed with or treated for a STI ^3^	149 (10%)	81 (9%)	68 (13%)
Prevalence of likely depression ^4^	218 (15%)	159 (17%)	59 (11%)
Providers	N = 40		
Demographics			
Age, med (IQR)	31 (27, 37)		
Sex—Female	16 (40%)		
Pharmacist/Pharmaceutical technologist	31 (78%)		
Level of training			
College Diploma	34 (85%)		
University Degree	5 (12%)		
Certificate	1 (2%)		
Years of training, med (IQR)	3 (3, 4)		
Own the pharmacy	17 (42%)		
Length of time in profession (in years), med (IQR)	6 (4, 10)		
Length of time worked at this pharmacy (in months), med (IQR)	33 (11, 60)		
Number of days work at this pharmacy per week, med (IQR)	6 (6, 7)		

^1^ Among those using contraception, 45% (431/954) were using a LARC and 33% (318/954) were using a non-LARC method. ^2^ In the past 3 months, have sex with more than 1 individual. ^3^ In the past 6 months. ^4^ According to Patient Health Questionnaire-2 (PHQ-2) score (≥3).

## Data Availability

The data that support the findings of this study are available on request from the corresponding author. The data are not publicly available due to privacy or ethical restrictions.
